# SOX2 in cancer stemness: tumor malignancy and therapeutic potentials

**DOI:** 10.1093/jmcb/mjy080

**Published:** 2018-12-05

**Authors:** Mahfuz Al Mamun, Kaiissar Mannoor, Jun Cao, Firdausi Qadri, Xiaoyuan Song

**Affiliations:** 1 Hefei National Laboratory for Physical Sciences at the Microscale, School of Life Sciences, University of Science and Technology of China, Hefei, China; 2 Oncology Laboratory, Institute for Developing Science & Health Initiatives (ideSHi), Dhaka, Bangladesh; 3 CAS Key Laboratory of Brain Function and Disease, CAS Center for Excellence in Molecular Cell Science, School of Life Sciences, University of Science and Technology of China, Hefei, China

**Keywords:** SOX2, cancer stem cells (CSCs), drug resistance, therapeutic potentials

## Abstract

Cancer stem cells (CSCs), a minor subpopulation of tumor bulks with self-renewal and seeding capacity to generate new tumors, posit a significant challenge to develop effective and long-lasting anti-cancer therapies. The emergence of drug resistance appears upon failure of chemo-/radiation therapy to eradicate the CSCs, thereby leading to CSC-mediated clinical relapse. Accumulating evidence suggests that transcription factor SOX2, a master regulator of embryonic and induced pluripotent stem cells, drives cancer stemness, fuels tumor initiation, and contributes to tumor aggressiveness through major drug resistance mechanisms like epithelial-to-mesenchymal transition, ATP-binding cassette drug transporters, anti-apoptotic and/or pro-survival signaling, lineage plasticity, and evasion of immune surveillance. Gaining a better insight and comprehensive interrogation into the mechanistic basis of SOX2-mediated generation of CSCs and treatment failure might therefore lead to new therapeutic targets involving CSC-specific anti-cancer strategies.

## Introduction

Cancer stem cells (CSCs), also known as tumor-initiating cells (TICs) or stem-like cancer cells, are a rare population of cells within cancer tissues with self-renewal capacity and ability to differentiate to diverse progenies of tumor cell (Clarke et al., 2006; Nguyen et al., 2012). They are also implicated in initiating metastasis and therapy resistance, thereby promoting tumor progression and disease recurrence (Chen et al. 2012; Valent et al. 2012; Kreso and Dick, 2014). Although the existence of CSCs remains controversial in physiological host settings, they have been functionally corroborated in many tumors on the basis of their ability to selectively engraft into immunocompromised mice upon transplantation at limiting dilutions, and to retain histopathological characteristics of the tumors of origins from where they had been isolated (Driessens et al., 2012; Schepers et al., 2012; Valent et al. 2012; Vanner et al. 2014; Woll et al. 2014). The presence of CSCs in human patients with the ability to generate an entire tumor, however, has yet to be elucidated. SOX2, also known as sex determining region Y (SRY)-box 2, is considered as one of the key-founding members of core pluripotency-associated transcription factors. Despite its active involvements in self-renewal and maintenance of stemness of embryonic- and neuronal stem cells (SCs), reprogramming somatic cells into induced pluripotent stem cells (iPSCs), and in regenerative medicine (Takahashi and Yamanaka, 2006; Niwa, 2007; Silva and Smith, 2008; Young, 2011); recent studies have demonstrated the oncogenic roles of SOX2 in cancers (Weina and Utikal, 2014; Wuebben and Rizzino, 2017).

## SOX2 as driving force for cancer stemness

**Figure 1:**
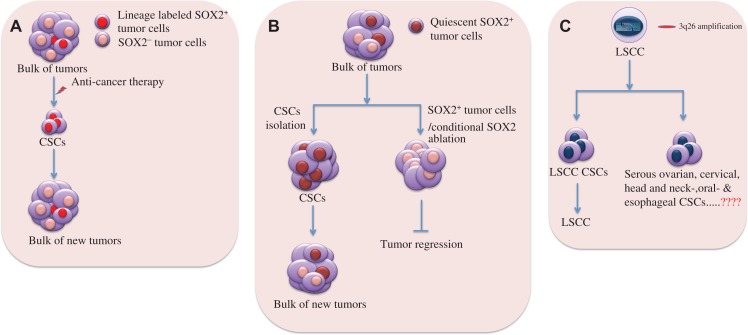
SOX2 as a key driver in cancer stemness. (**A** and **B**) SOX2^+^ tumor cells are relatively quiescent, avoid chemotherapy, reinitiate tumor growth, and give rise to differentiated cell progenies that recapitulate primary tumor compositions. (**A**) Anti-tumor therapy kills most of the tumor cells, leaving behind SOX2^+^ tumor cells that serve as CSCs for tumor regrowth. Lineage tracing experiments showed that developmental hierarchies were preserved, and SOX2^+^ tumor cells were therapy resistant and responsible for tumor progression. (**B**) The representative image shows enrichment of (quiescent) SOX2^+^ tumor cells from tumor masses, which seed for new tumors containing both SOX2^+^ and SOX2^−^ tumor cells (left). Lineage-specific ablation of SOX2^+^ tumor cells or conditional SOX2 deletion leads to tumor regression (right). (**C**) High SOX2 and PRKCI expressions in LSCC gain via 3q26 chromosomal amplification. Coordinated overexpression of both of these proteins is attributed to LSCC stemness and enhanced tumorigenicity. Since 3q26 copy number gains are the most frequently occurring mutation in SOC, cervical, head and neck, oral, and esophageal carcinomas, it might be involved in the generation of respective CSCs.

Amidst the core regulators of stemness (e.g. OCT4, SOX2, and NANOG), SOX2 expression has been identified in myriad diversities of cancers with poor disease prognosis (Tam and Ng, 2014; Weina and Utikal, 2014; Wuebben and Rizzino, 2017). High level of SOX2 expression is a key in conferring stem cell-like phenotypes to more than a dozen of tumors (Table 1). Independent studies by Vanner et al. (2014) and Boumahdi et al. (2014) reported that SOX2-expressing (SOX2^+^) tumor cells could drive cancer malignancy by serving as the founding population with the ability to initiate and propagate tumor growth and give rise to the diversity of differentiated cell progenies in different cancer types (Figure 1A and B). Their genetic fate mapping and limiting dilution transplantation assay demonstrated SOX2 as a cancer stem cell marker in medulloblastoma and skin squamous cell carcinoma (SCC). Medulloblastoma growth paralleled a developmental stem cell hierarchy driven by quiescent SOX2^+^ tumor-initiating cells and therapeutic interference with the SOX2^+^ cells could stop tumor growth. Similarly, lineage ablation of SOX2^+^ tumor cells and conditional deletion of SOX2 in pre-existing skin papilloma and SCC could cause tumor regression. SOX2 expression had been shown as a potential CSC marker in bladder cancers (BCa) where SOX2-expressing cells could seed the BCa, and lineage-specific ablation of SOX2-expressing cells enhanced tumor regression (Zhu et al., 2017). SOX2^+^ cells in ovarian cancers could retain *in vivo* tumor-initiating capability and were responsible for therapy resistance and tumor aggressiveness (Bareiss et al., 2013). In high-grade gliomas, SOX2 was frequently overexpressed and essential for maintenance of glioma stem cells to reinitiate and drive tumorigenicity (Gangemi et al., 2009; Ikushima et al., 2009; Hägerstrand et al., 2011). These observations are in consistent with the previous studies on different cancer types, including melanomas (Santini et al., 2014), osteosarcomas (Basu-Roy et al., 2012), head and neck SCC (HNSCC) (Lee et al., 2014; Keysar et al., 2017), breast cancer (Lengerke et al., 2011; Leis et al., 2012; Gupta et al., 2018), squamous cancer (Siegle et al., 2014), colorectal cancer (CRC) (Lundberg et al., 2016), cervical cancer (Liu et al., 2014), pancreatic cancer (Herreros-Villanueva et al., 2013), lung cancer (Xiang et al., 2011; Chen et al., 2012; Singh et al., 2012), and gastric cancer (Tian et al., 2012), further highlighting the critical roles of SOX2 in seeding and refueling unconstrained CSCs. This also hints that SOX2 promoter was epigenetically suppressed in differentiated tumor cells. It is likely that aberrant activation of SOX2 promoter upon epigenetic changes within tumor microenvironment could cause a subpopulation of tumor cells to shift towards a cancer stem-like phenotype (Figure 1A and B).

**Table 1:** Role of SOX2 in cancer stemness.

Cancer type	SOX2-expressing cells	CSCs/TICs	SOX2 expression in CSC	SOX2 depletion	Genetic means for SOX2 depletion	References
Glioma	Yes	GBM CSC/glioma stem cells/GBM neurosphere		Halts CSC generation	shRNA	Jeon et al. (2011)
	Yes	GBM TIC	High	Halts TIC	shRNA	Gangemi et al. (2009)
	Yes	GBM neurosphere	High	Reduces GBM CSC	siRNA	Hägerstrand et al. (2011)
	Yes	Glioma initiating cells	High	Reduces neurosphere	siRNA	Ikushima et al. (2009)
	Yes	GBM CSC	High	Abolishes CSC phenotypes	shRNA	Berezovsky et al. (2014)
Lung cancers	Yes	LSCC stem-like cells	High	Impairs oncosphere proliferation, produced small and disorganized oncospheres	shRNA	Justilien et al. (2014)
	Yes	NSCLC stem cells	High	Lose cancer stemness	siRNA	Singh et al. (2012)
	Yes	NSCLC CSC	High	Suppress tumor growth & metastasis	siRNA	Xiang et al. (2011)
Breast cancers	Yes	Mammosphere	High	Reduce CSC	siRNA	Piva et al. (2014)
	Yes	Breast CSC	High	Diminishes CSC	siRNA	Mukherjee et al. (2017)
Medulloblastoma	Yes	Medulloblastoma propagating cells (MPCs)	High	NA	NA	Vanner et al. (2014)
Papilloma & skin SCC	Yes	SCC CSC	High	Tumor regression	SOX2^+^ lineage ablation & conditional SOX2 deletion	Boumahdi et al. (2014)
Colorectal cancers (CRC)	Yes	CRC CSC	High	NA	NA	Lundberg et al. (2016)
HNSCC	Yes	HNSCC CSC	High	Reduces self-renewal capacity	shRNA	Lee et al. (2014)
	Yes	HNSCC CSC	High	NA	NA	Keysar et al. (2017)
Sarcomas	Yes	Osteospheres	High	Fail to form osteospheres	shRNA	Basu-Roy et al. (2012)
Pancreatic ductal adenocarcinoma (PDAC)	Yes	PDAC CSC	High	Reduces CSC generation	shRNA	Herreros-Villanueva et al. (2013)
Serous ovarian carcinoma (SOC)	Yes	SOC CSC	High	Reduces sphere formation	shRNA	Bareiss et al. (2013)
Melanoma	Yes	Melanoma-initiating cells	High	Reduces MIC self-renewal capacity	shRNA	Santini et al. (2014)
Gastric cancers	Yes	Gastric cancer stem-like cells	High	Reduces colony formation	siRNA	Tian et al. (2012)
Cervical cancers	Yes	Cervical cancer CSC	High	NA	NA	Liu et al., (2014)
Bladder cancers	Yes	Bladder cancer CSC	High	Tumor regression	SOX2^+^ lineage ablation	Zhu et al. (2017)

Although high levels of SOX2 expression had been found without any genetic mutations in most of the cancers, which indicates the association of epigenetic events, certain cancers could exhibit high levels of SOX2 expression due to gene amplification. TCGA profiling (http://www.cbioportal.org) of RNA datasets of 840 cases with human lung squamous cell cancers (LSCC) estimated that SOX2 was amplified in 44.6% (*n* = 375) cases. Actually chromosome 3q26 amplification causes the most prevalent copy number gains and this phenomenon drives coordinated overexpression of SOX2 and PRKCI (a protein kinase Ci that phosphorylates SOX2) in majority of human LSCC, activates PRKCI–SOX2–HHAT signaling axis, and ultimately leads to the establishment of a stem-like, LSCC tumor-initiating cell phenotype (Balsara et al., 1997; Justilien et al., 2014). Other than the lung cancers, the chromosome 3q26 copy number gain was also the most frequently occurring genetic alterations in serous ovarian carcinoma (SOC) (Sugita et al., 2000), cervical (Sugita et al., 2000), head and neck (Snaddon et al., 2001), oral (Lin et al., 2005), and esophageal (Imoto et al., 2001) tumors. Association of 3q26 amplification with PKCi–SOX2–HHAT signaling axis might also play a regulatory role by imparting stem-like phenotypes in different tumor types harboring these chromosomal alterations (Figure 1C). The most common somatic mutations in LSCC could lead to inactivation of the tumor suppressors, such as LKB1, PTEN, TP53, and RB. In LSCC mouse model mimicking human LSCC, Mukhopadhyay et al. (2014) showed that lung-specific Lkb1 loss in association with enforced overexpression of SOX2 could drive formation of tumors with solely squamous morphology (LSCC), while the expression of SOX2 in the context of Trp53 loss, either alone or in co-ordination with Rb loss, could induce lung adenocarcinoma (LADC). These findings suggest that SOX2 regulates differentiation of tumor types by promoting tumor formation depending upon the loss or inactivation of tumor suppressors. Similarly, certain mouse models bearing various combinations of genetic lesions that were predominantly found in human LSCC had revealed the determinative role of SOX2 in squamous lineage restriction and proved SOX2 as a key oncogenic LSCC driver. They described that SOX2 overexpression upon simultaneous loss of PTEN and CDKN2AB could lead to the development of LSCC from basal, alveolar type 2 (AT2), and club cells. Hence, SOX2 overexpression drives PTEN- and CDKN2AB-deficient heterogeneous lung tumors into LSCC regardless of cell origins through lineage restriction. SOX2 overexpression alone in lung can give rise to hyperplasia and tumors of the adenocarcinoma lineage (e.g. LADC) either from AT2 or club secretory cells (Lu et al., 2010). These studies concluded that SOX2 overexpression together with other cooperating mutations was determinative in driving transformation of different cell types in lung towards LSCC or LADC, thereby defining the role of SOX2 in lineage-specific survival mechanism of cancers via initiation of multiple genealogical new tumors. It might also hold the possibility of existence of CSCs in between the transition from LSCC to LADC, or from basal, AT2 and club cells to LSCC, thus, pointing to the role of SOX2 in generation of CSCs in SOX2^+^ tumors upon genetic insults.

These evidences support SOX2 as a prospective biomarker for cancer stemness in this sense that even if there were numerous CSC markers, such as cell surface marker CD15 (stage-specific embryonic antigen 1), very few of them had been demonstrated to actively promote stem-like properties. Also, lineage-specific ablation of SOX2^+^ tumor cells or depletion of SOX2 expression could retard tumorigenicity by disconnecting the network system that favored generation of CSCs, thereby, preventing further tumor regrowth (Figure 1). This idea also signals to the notion that both epigenetic and genetic dysregulations of SOX2 might contribute to high plasticity and heterogeneity of neoplastic cells by facilitating formation of CSCs, as these cells were known to generate intra-tumor heterogeneity (Tang, 2012; Prasetyanti and Medema, 2017).

## SOX2 in context of refractoriness to anti-cancer therapies and clinical relapse

Tumor cells can become resistant to anti-cancer drugs in several ways and this makes the task of finding a solution to this problem more difficult. They may enter a temporary ‘drug-tolerant state’ that could help them to survive and develop resistance to the drug. The penetrance of CSCs in addition to their long-term self-renewal ability is attributed to resistance to conventional anti-cancer therapies (Dean et al., 2005; Clarke et al., 2006). The surviving therapy-resistant CSCs have potential to serve as the precursors of newly formed tumor masses, eventually leading to clinical relapse (Bao et al., 2006; Li et al., 2008; Zhou et al., 2009) (Supplementary Figure S1). Apart from its role in imparting stem-like characteristics to cancers, SOX2 has been implicated in developing resistance to chemotherapeutics used in common clinical set up as discussed briefly in this section (Bareiss et al., 2013; Piva et al., 2014; Mu et al., 2017; Mukherjee et al., 2017).

### Gliomas

SOX2 expression had been detected in most of the gliomas (Gangemi et al., 2009; Annovazzi et al., 2011; Guo et al., 2011; Vasquez et al., 2017). Several studies reported high levels of SOX2 expression in glioblastoma multiforme (GBM; WHO IV) than in the low-grade gliomas (LGGs) (Annovazzi et al., 2011; Guo et al., 2011; Vasquez et al., 2017). GBM, one of the top ranked aggressive brain malignancies, is considered as the most prevalent brain tumor accounting for approximately 65% of all primary brain tumors, and is characterized by poor survival rate with only 10% of patients surviving 5 years (Stupp et al., 2009; Johnson and O’Neill, 2012). Even after successful surgical tumor resection followed by concurrent radiation therapy with temozolomide (TMZ; oral methylation chemotherapy) and subsequent follow-up treatment with additional adjuvant TMZ, GBM showed a very poor outcome with almost 100% recurrence. This is due to the existence of ‘glioma stem cells (GSCs)’ having capability of tumor initiation, self-renewal and aberrant differentiation. GSCs had been responsible for disease progression as a consequence of remarkable resistance to chemotherapy and irradiation, which are the first-line treatment options for the patients with malignant gliomas (Bao et al., 2006; Vescovi et al., 2006; Wang et al., 2010; Auffinger et al., 2015; Zhou et al., 2015). Besides, SOX2^+^ tumor cells were enriched in gliomas that could relapse following radiation therapy or chemotherapy with TMZ, and it was evinced that glioma cell population with CD133^+^ (an important marker for GSC) had higher levels of SOX2 expression (Bao et al., 2006; Auffinger et al., 2015). Additionally, SOX2 was ubiquitously expressed in almost all GBM neurosphere cell cultures (Gangemi et al., 2009; Fang et al., 2011; Hägerstrand et al., 2011; Bulstrode et al., 2017). Despite the fact that resistance to TMZ was associated with the abundant expression of O6-methylguanine-DNA-methyltransferase in CD133 positive GBM stem cells (Liu et al., 2006), a major cause of chemoresistance in GSCs was the activation of multi-drug resistance ATP-binding cassette (ABC) transporter genes (Dean et al., 2005), which had higher levels of expression in CSCs than their differentiated counterparts (Nakai et al., 2009). In GBM, the glioma cells acquired stemness upon induction of SOX2 by inhibitor of differentiation 4 (ID4) and showed resistance to anti-cancer drugs BCNU (1,3-bis(2-chloroethyl)-1-nitrosourea) by upregulating the expression of ABCC3 and ABCC6, indicating that SOX2 played a pivotal role in controlling ABC transporter-mediated chemoresistance in both patients-derived and induced GSCs (Jeon et al., 2011). Furthermore, activation of PI3K/Akt signaling upon PTEN loss conferred chemoresistance to GSCs by enhancing ABCG2 activity (Bleau et al., 2009). SOX4-mediated SOX2 expression could activate TGF-β signaling and maintain GSC stem-like properties and tumorigenicity. Targeting TGF-β–Sox4–SOX2 axis by TGF-β signaling inhibitor could impair GSC tumorigenicity (Ikushima et al., 2009). Gangemi et al. (2009) also observed that SOX2 depletion in GSCs could promote differentiation and loss of stemness and tumorigenicity. Hägerstrand et al. (2011) identified a SOX2-dependent subset of tumor- and sphere-forming glioblastoma cells, which possessed higher capacity to form xenograft tumors and neurospheres and displayed low or no sensitivity to mono-treatment with PDGF (platelet-derived growth factor)-receptor inhibitor (e.g. imatinib) and insulin-like growth factor-1 (IGF-1) receptor inhibitor (e.g. NVP-AEW541). Thus, therapy-resistant SOX2^+^ tumor cells seem to serve as precursors for GSCs to cause clinical relapse.

### Lung cancers

SOX2, the most frequently altered gene in human SCC (skin, lung, and esophageal carcinomas), is amplified in >20% and overexpressed in 60%–90% of tumors. SOX2 is also very often expressed in early stage SCC. These all suggest that deregulated SOX2 expression might be an initiating event in development of SCC (Bass et al., 2009; Hussenet et al., 2010; Brcic et al., 2012). Indeed, human LSCC representing 30% of lung cancers contained stem-like cells responsible for initiation, maintenance, metastasis, and relapse of lung tumor (Eramo et al., 2008; Justilien et al., 2014). In non-small cell lung cancer (NSCLC), SOX2 expression was reported to be significantly higher (*P* = 0.01) in metastasized tumors than in primary site or lower stage tumors and such higher level of SOX2 expression accounted for maintaining self-renewal and expansion of NSCLC stem cells through EGFR/Src/Akt signaling. SOX2 suppression by RNAi or abrogation of EGFR, Src, or Akt signaling through EGFR tyrosine kinase inhibitors Gefitinib, Erlotinib, or BIBW2992 or Src inhibitor Dasatinib could result in curbing stem-like properties and regrowth of tumor. In NSCLC, SOX2 expression could promote cell proliferation and survival by an increase in emergence of acquired resistance to commonly used drugs, namely cisplatin and paclitaxel through activation of oncogenic EGFR and BCL2L1 signaling (Chou et al., 2013). Silencing SOX2 in TICs could suppress growth and metastasis of lung cancers (Xiang et al., 2011). Following treatment of NSCLC with the EGFR inhibitor erlotinib, SOX2 expression was reported to be higher in NSCLC harboring EGFR mutations than in controls and more likely such tumors could develop resistance to the drug by repressing pro-apoptotic BH3-only genes, such as BIM and BMF. Reduction of SOX2 expression could cause a substantial decrease in the number and rate at which the resistant sub-clones of EGFR-mutant cells appeared due to continual treatment with erlotinib, thereby displaying the role of SOX2 in the emergence of stably acquired resistance (Rothenberg et al., 2015). In addition, SOX2 expression could also contribute to the emergence of drug resistance through ‘lineage specific survival mechanism’ (Lu et al., 2010; Mukhopadhyay et al., 2014; Ferone et al., 2016). Together, these findings identify SOX2 as a promising target for therapeutic interventions in lung cancer.

### Breast cancers

SOX2 expression has been reported to associate with the malignancy of tumors in breast cancer (Lengerke et al., 2011; Leis et al., 2012; Piva et al., 2014). High levels of SOX2 expression with endocrine treatment failure and poor relapse-free survival were observed in a cohort of ER-positive patients with breast cancer who had received tamoxifen therapy. In this cohort, 40% patients responded to endocrine therapy (*n* = 22) and 41.8% failed to respond to endocrine therapy (*n* = 23), resulting in 41.8% recurrence after therapy failure (*n* = 23) (Piva et al., 2014). Importantly, there was a significant increase in SOX2 expression in the recurrent lesions compared to the primary tumors. The development of tamoxifen resistance was accompanied by an elevation of SOX2 expression and loss of ER transcriptional activity, leading to activation of Wnt signaling and enrichment of the CSC population. On the contrary, a reduction in endogenous SOX2 levels could cause a decrease in the proportion of the rare population of stem cells and enhance the sensitivity to tamoxifen *in vitro* and *in vivo*. Notably, breast stem/progenitor cells lack or express low levels of ER (Clayton et al., 2004; Liu et al., 2008). Higher levels of SOX2 expression had also been investigated in a cohort of patients with triple negative breast cancer (TNBC; ER^−^, PR^−^, HER2/neu^−^) (*n* = 30) who previously received chemotherapy. An increase in SOX2 expression could result in an elevation of ABCG2 and TWIST1 in patients-derived CSCs and MDA-MB-231 CSCs, thereby enhancing chemoresistance to paclitaxel along with exalted invasiveness via epithelial to mesenchymal transition (EMT) (Mukherjee et al., 2017). TWIST1 had been demonstrated as a key regulator of invasiveness and EMT pathway (Nuti et al., 2014). TWIST1 expression had also been involved in chemoresistance (Wang et al., 2004; Li et al., 2009) and stem-like properties in breast cancer (Liang et al., 2015). SOX2 plays an important role in carcinogenesis of early stage breast tumors and possibly promotes tumor metastasis as metastatic lymph nodes were reported to enrich in SOX2 expression (Leis et al., 2012).

### HNSCC

SOX2 expression was associated with poor prognosis in patients with HNSCC (Du et al., 2011; Tang et al., 2013; Schrock et al., 2014). CSCs in patients with HNSCC had been closely connected to tumor invasion and metastasis and tumor regrowth could occur as a consequence of their resistance to conventional chemo- and radiotherapy (Zhang et al., 2012; Chen et al., 2013a, b). Lee et al. (2014) showed that upregulation of ABCG2 in HNSCC stem cells as a result of higher levels of SOX2 expression was associated with accelerated chemoresistance to cisplatin. Ablation of SOX2 in the CSCs of the patients with HNSCC attenuated their ability of self-renewal, chemoresistance, invasion, and *in vivo* tumorigenicity in mouse model. They demonstrated that SOX2^+^ tumors (45%, *n* = 69 patient samples) with higher levels of SOX2 expression were remarkably associated with disease relapse with a 4.7-fold higher risk in contrast to the patients with lower SOX2 expression profile. Keysar et al. (2017) explored the role of SOX2 in patient-derived xenografts (PDXs) (*n* = 10) of HNSCC model and demonstrated that SOX2 protein levels were dramatically higher in ALDH^+^ (ALDH1A1) CSCs of PDX origin and accountable for conferring enhanced stemness and drug resistance (docetaxel) to HNSCC CSCs, which could recapitulate the heterogeneity of the original tumor via their ability to asymmetric division, thus resulting in sustained tumor growth. They concluded that an increase in translation of SOX2 in HNSCC was favored by upregulation of PI3K signaling that was frequently activated in HNSCC by PIK3CA amplification or mutation (Yuan and Cantley, 2008).

### Sarcomas

SOX2 was linked to enhanced tumorigenicity in pediatric sarcomas (Skoda et al., 2016), and it was highly expressed in human and murine osteosarcomas (Basu-Roy et al., 2012). SOX2 could act as a survival factor and impart CSC properties to osteosarcomas by antagonizing pro-differentiation Wnt signaling pathway (Basu-Roy et al., 2012). SOX2 was further demonstrated to interfere with tumor-suppressive Hippo pathway to maintain CSCs in osteosarcomas. The SOX2-Hippo regulatory circuit remained conserved in multiple SOX2-dependent cancers like GBMs (Basu-Roy et al., 2015). These observations might provide an explanation for the poor response of osteosarcomas to chemotherapy, as osteospheres had been reported to be refractory to chemotherapeutic drugs (Fujii et al., 2009).

### Pancreatic ductal adenocarcinoma

Pancreatic ductal adenocarcinoma (PDAC) had been reported to be one of the most notorious malignancies having a median survival of <1 year for patients with locally advanced or metastatic disease (Kanji and Gallinger, 2013). SOX2 expression increased remarkably from ~20% in pre-malignant PanIN3 lesions to nearly 60% of poorly differentiated PDAC during course of tumor progression (Sanada et al., 2006). SOX2 also could act as a molecular rheostat in SOX2^+^ PDAC for their growth, tumorigenicity and responsiveness to anti-cancer drugs (Wuebben et al., 2016). Herreros-Villanueva et al. (2013) further uncovered the role of SOX2 as a CSC maker in PDAC (SOX2^+^ PDAC) and indicated that aberrant expression of SOX2 could contribute to proliferation, generation of stem-like properties and dedifferentiation of PDAC by controlling EMT phenotypes. Targeting SOX2^+^ PDAC, therefore, could be a promising therapeutic strategy to root out CSCs in PDAC to prevent cancer progression, drug resistance and recurrence.

### SOC

Higher levels of SOX2 protein expression (10%–60%) in SOC was associated with tumor aggressiveness in terms of histopathological and clinical manifestations (Zhang et al., 2012; Pham et al., 2013), indicating that SOX2 might have played a pivotal role in maintenance of stem-like features of SOC. Indeed, SOX2 expression in SOC propagating cells enabled their selective survival to conventional chemotherapies and promoted the *in vivo* tumorigenicity. The SOX2^+^ SOCs contributed to therapy resistance to staurosporine, carboplatin, cisplatin and paclitaxel and disease relapse in the patients with ovarian cancer through induction of cancer stemness and apoptosis resistance (Bareiss et al., 2013).

### CRC

SOX2 expression is related to lymph nodes and distant metastases in CRC (Neumann et al., 2011) and enhanced invasiveness (Han et al., 2012) with poor disease prognosis (Lundberg et al., 2014). This might be attributed to SOX2^+^ tumor cells that induce a cellular stem cell state in human CRC with low levels of CDX2 expression (Lundberg et al., 2016).

### Melanoma

Approximately 50% of melanomas express SOX2 (Laga et al., 2010; Chen et al., 2013a, b) and SOX2 depletion is associated with reduced growth and invasiveness of melanoma (Laga et al., 2010; Girouard et al., 2012). Melanoma-initiating cells (MICs) are considered to be resistant to conventional chemotherapeutics (Frank et al., 2005). SOX2 is a critical factor for maintenance of self-renewal capacity of MICs and their tumorigenicity (Santini et al., 2014).

### Gastric cancers

CSC in gastric cancers had been reported recently (Chen et al., 2012; Tian et al., 2012) and SOX2 had been implicated in maintaining stem-like properties of gastric cancer cells and enhanced chemoresistance to cisplatin or Adriamycin, possibly via upregulation of ABC drug transporters (MRP2 and MDR1) (Tian et al., 2012).

### Medulloblastoma

Quiescent SOX2^+^ tumor cells seed for medulloblastoma-propagating cells (MPCs) and cause relapse in SHH medulloblastoma. The MPCs are highly resistant to cytarabine or vismodegib (Vanner et al., 2014).

### Miscellaneous cancers

Elevated SOX2 expression in epithelial TICs (e.g. lung and breast cancers) had been described to be responsible for evasion of complement surveillance (Chen et al., 2017). In prostate cancers, SOX2 could promote resistance to antiandrogen therapy (e.g. enzalutamide) by turning on lineage plasticity (Mu et al., 2017). SOX2 was expressed in pre-neoplastic and invasive bladder tumors, whereas it was absent in normal urothelial cells and SOX2 could facilitate tumor invasiveness through generation of stem cells in bladder cancer (Zhu et al., 2017).

## Mechanistic links to the therapeutic resistance

**Figure 2:**
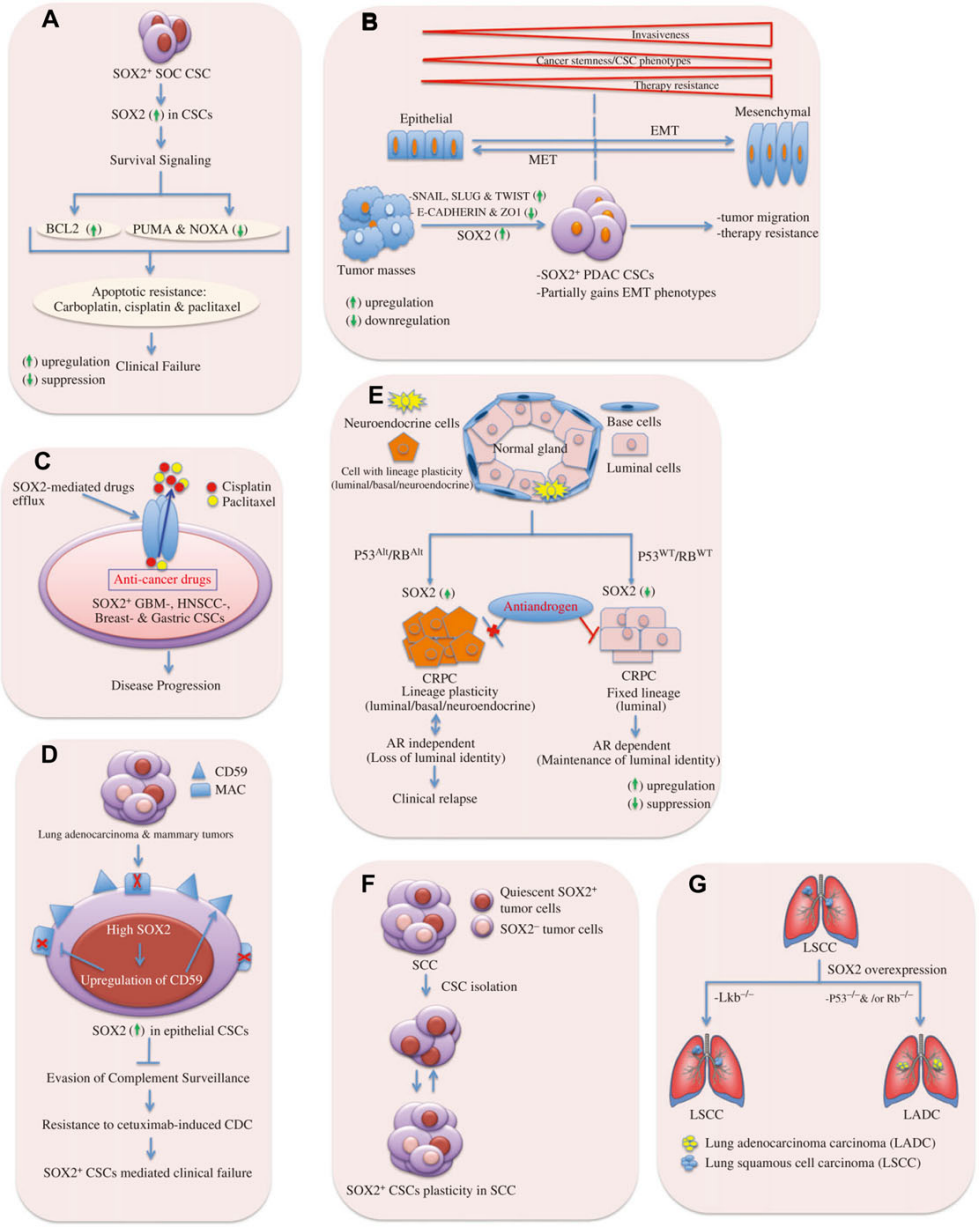
Mechanistic links to SOX2-dependent CSC-mediated clinical relapse. (**A**) SOX2 mediates survival signal to CSCs. In SOC CSCs, elevated SOX2 expression is associated with upregulation of anti-apoptotic factor BCL2 and suppression of pro-apoptotic proteins PUMA and NOXA. This provides survival signal to persist under anti-cancer drugs as carbolatin, cisplatin, or paclitaxel, and thus, enhancing apoptotic resistance. (**B**) CSCs gain partial EMT phenotypes facilitating tumor malignancy due to SOX2 expression. It illustrates the extent of invasiveness, tumor-initiating ability, and a change in degree of drug resistance across the spectrum of EMT-program activation. Tumor invasiveness and drug resistance increase upon gaining complete EMT phenotypes. The cancer stemness or tumor-initiating ability of carcinoma cells is influenced by the level of EMT-program activation and it peaks at an intermediate level of EMT. Indeed, extensive EMT activation is usually detrimental to tumor-initiating ability. The drug resistance of carcinoma cells also seems to be maximal at an intermediate level of EMT-program activation, but plateaus (rather than declines) with further activation of this program. In pancreatic adenocarcinoma (PADC), SOX2 expression imparts partial EMT-like phenotypes to PADC CSCs via upregulation of the EMT master regulators SNAIL, SLUG, and TWIST, and it is shown that SOX2 cannot induce fully EMT program in PADC. Thus, SOX2 contributes to EMT-mediated tumor malignancy. MET, mesenchymal-to-epithelial transition. (**C**) SOX2 mediates drug efflux in CSCs. SOX2 in CSCs enhances the expression of ABC drug transporters that can effectively efflux anti-cancers drugs (e.g. cisplatin, and paclitaxel) from glioblastoma (GBM), breast cancer, gastric cancer, and HNSCC CSCs through hydrolysis of ATP. Thus, CSCs acquire resistance to therapy and cause clinical failure. CSCs are likely to share many properties of normal stem cells providing an opportunity for a long lifespan, e.g. they remain relative quiescence, show resistance to drugs, and efflux toxins through expression of ABC transporters. This points to the tumors having built-in population of drug-resistant pluripotent cells that can survive chemotherapy and repopulate the tumor. (**D**) SOX2 mediates evasion of complement surveillance by CSCs. High SOX2 expression in epithelial CSCs causes upregulation of CD59, which in turn leads to inhibition of membrane attack complex (MAC). Thus, CSCs avoid complement attacks and show enhanced resistance to CDC. (**E**) SOX2 promotes lineage plasticity in p53^−/^^−^ and Rb^−/^^−^ prostate cancers. SOX2 is responsible for anti-androgen resistance in castration-resistant prostate cancers of adenocarcinoma histology (CRPC-adeno) due to TP53 and RB1 alterations (TP53^Alt^, RB1^Alt^) compared to those with WT TP53 and RB1 (TP53^WT^, RB1^WT^). High SOX2 expression leads to anti-androgen drug resistance (e.g. enzalutamide) in CRPC-adeno upon loss of tumor suppressor genes p53 and Rb. Luminal identity is characterized by the presence of androgen receptor (AR). The tumors can develop resistance to the anti-androgen drug by a phenotypic shift from androgen receptor (AR)-dependent luminal epithelial cells to AR-independent basal-like cells with mixed phenotypes (luminal/basal/neuroendocrine cells). (**F**) SOX2 provides lineage plasticity to CSCs. In skin SCC, SOX2^+^ tumor-propagating cells give rise to both SOX2-expressing and SOX2-negative tumor cell progenies, thus, imparting plasticity to CSCs. (**G**) SOX2 involves in lineage-specific survival mechanism. SOX2 overexpression gives rise to LSCC upon loss of the tumor suppressor Lkb or LADC upon loss of p53 and/or Rb.

Emergence of resistance to chemo/radio-therapy in cancer cells could be acquired by a range of mechanisms including switching on tumor plasticity and EMT programs, removal of drug molecules from the cells by ABC drug transporters system, activation of pro-survival and anti-apoptotic signaling, activation of pathways responsible for lineage-specific survival, mutation or overexpression of the drug target, and evasion of immune surveillance (Figure 2) (Dean et al., 2005; Morel et al., 2008; Chen et al., 2009; Ikushima et al., 2009; Bareiss et al., 2013; Holohan et al., 2013; Ye et al., 2015; Malladi et al., 2016; Shibue and Weinberg, 2017).

Typically, SOX2 is involved in major mechanisms ascribed to the phenomena of therapeutic resistances in cancers, which ultimately lead to clinical relapse. For instance, SOX2 is credited to catalyzing pro-survival and anti-apoptotic signaling in diverse range of cancers, such as SOX2 expression develops resistance to commonly used drugs in lung cancer (e.g. erlotinib, cisplatin, paclitaxel) by suppressing pro-apoptotic BH3-only genes, namely BIM and BMF (Rothenberg et al., 2015) and through activation of oncogenic EGFR and BCL2L1 signaling (Chou et al., 2013). In SOC, SOX2^+^ cells could contribute to therapy resistance (e.g. staurosporine, carboplatin, cisplatin and paclitaxel) by inducing CSCs and upregulating anti-apoptotic factor BCL2 and by a reduction in the expression of pro-apoptotic proteins, such as PUMA and NOXA (Figure 2A) (Bareiss et al., 2013).

Promotion of CSC-mediated cancer relapse occurs via activation of epithelial-to-mesenchymal transition (EMT) program and expression of ABC drug transporters (Dean et al., 2005; Ye et al., 2015; Shibue and Weinberg, 2017). In a number of cancers, only CSC-enriched subpopulation could exhibit the aspects of EMT-program activation with an increase in tumor initiating and migrating capacities (Mani et al., 2008; Morel et al., 2008; Chen et al., 2009; Ikushima et al., 2009). In CSCs, SOX2-mediated drug resistance was acquired through activation of EMT pathway in CRC (Han et al., 2012), HNSCC (Lee et al., 2014), PDAC (Herreros-Villanueva et al., 2013), and breast cancers (Mukherjee et al., 2017). In pancreatic cancers, SOX2^+^ PDAC cells could gain cancer stem-like pluripotent potentials through partial EMT phenotypes: upregulation of EMT master regulators (e.g. SNAIL, SLUG, and TWIST), and downregulation of epithelial markers (e.g. E-cadherin and ZO1). Although SOX2 possesses the ability to drive dedifferentiation and induction of the expression of certain EMT markers, it is unable to confer a full mesenchymal phenotype, which might be suggestive of incomplete overlapping transcriptional programs underlying CSCs and EMT (Figure 2B) (Herreros-Villanueva et al., 2013). Likewise, resistance properties to anti-tumor therapy are associated with SOX2-mediated activation of multidrug resistance ABC transporter genes (Dean et al., 2005) because their protein products are able to efflux drugs across the cell membrane by utilizing ATP in glioblastoma (Jeon et al., 2011), HNSCC (Lee et al., 2014), gastric cancers (Tian et al., 2012), and breast cancers (Figure 2C) (Mukherjee et al., 2017).

Deregulated Wnt/β-catenin signaling had been implicated in mediating therapy resistance to breast cancers (Chen et al., 2007; Forget et al., 2007). Development of tamoxifen resistance had been reported to occur due to SOX2-dependent activation of Wnt/β-catenin signaling pathway in breast cancers (Piva et al., 2014).

The aggressiveness of CSCs might occur as a result of their ability to avoid human immune complement system. The recent finding by Malladi et al. (2016) revealed that latency-competent cancer cells (LCC, stem-like cancer cell) isolated from early stage breast and lung cancers expressed SOX2 and SOX9, which were essential for their survival in host organs under immune surveillance by natural killer cells, and for metastatic outgrowth under permissive conditions by attenuating WNT signaling. This study gives prominence to SOX2/SOX9 for evolution of metastasis-initiating cells in multiple host tissues and for entry into a quiescent stage by which a minority of LCC cells can evade NK cell surveillance. This observation could provide a link to latency metastasis showed by disseminated tumor cells (DTCs), which remain quiescent, evade immunity, retain tumor-initiating capacity, and evolve into an aggressive metastatic state (Massague and Obenauf, 2016). Clinically, many patients who were considered as disease-free after receiving cancer treatment might often harbor thousands of DTCs in bone marrow and other organs, thereby heading towards clinical relapse (Braun et al., 2005). In breast and lung epithelial CSCs, the upregulation of CD59 as a result of high levels of SOX2 expression was involved in resistance to cetuximab-induced complement-dependent cytotoxicity (CDC) by evading complement surveillance. This was also associated with enhanced EGFR expression; thereby conferring tumor-propagating cells a growth signal for their survival in the tumor microenvironment (Figure 2D) (Chen et al., 2017).

CSC progenies can manifest diverse plasticity because of their phenotypic and functional heterogeneity, for instance, some cancers having lineage plasticity can bypass targeted therapies through acquisition of phenotypic characteristics of a cell lineage whose survival no longer depends on the drug target (Tang, 2012). More recently, Mu et al. (2017) reported SOX2 to enhance resistance to antiandrogen therapy in TP53- and RB1-deficient human prostate cancer models by switching lineage plasticity. The tumors could develop resistance to the antiandrogen drug (e.g. enzalutamide) by a phenotypic shift from androgen receptor (AR)-dependent luminal epithelial cells to AR-independent basal-like cells. This lineage plasticity was achieved via loss of TP53 and RB1 function and by an increase in SOX2 expression, which could be backed by restoring TP53 and RB1 function or by silencing expression of SOX2. This might explain one possible pathway in which SOX2^+^ tumor cells could exhibit an enhanced resistance to chemotherapeutics and long-term tumor-propagating capability (Figure 2E). Boumahdi et al. (2014) provided further evidence in support of reversible plasticity between tumor-initiating cells and their differentiated progenies by demonstrating that SOX2^+^ tumor epithelial cells could give rise to tumors that might contain both SOX2^−^ and SOX2^+^ tumor epithelial cells. Thus, the conversion between CSCs and their non-stem cell progenies appears reversible in melanoma (Figure 2F). Vanner et al. (2014) also demonstrated that enrichment of SOX2^+^ tumor cells in medulloblastoma following anti-mitotic chemotherapy (e.g. cytarabine) or sonic hedgehog pathway inhibitor (vismodegib) created a reservoir for further tumor regrowth. The ability of SOX2^+^ medulloblastoma-propagating cells to stay quiescent and resist anti-mitotic drugs confers an advantage for survival under severe selection pressure. This may reflect at least one mode by which tumor-initiating cells are protected from certain cancer therapies. Quiescence is a defining characteristic of many somatic stem cells (Li and Clevers, 2010). Self-renewing quiescent cancer cells had been identified in several cancer malignancies (Guan et al., 2003; Saito et al., 2010) and were often resistant to conventional chemo- and radiation therapy, thus acting as a reservoir for tumor re-initiation. SOX2 also plays a crucial role in GBM malignancy by controlling the expression of key genes involved in both cancer stem-like and differentiated cells and perpetuates plasticity for bidirectional conversion between these two states (Berezovsky et al., 2014). Balca-Silva et al. (2017) demonstrated that SOX2 was expressed at high levels in GBMs compared to the lower-grade gliomas, and responsible for plasticity of GBM stem cells that indicated the inter-conversion between non-GSCs and GSCs states. Genetic lineage tracing experiments of primary tumors in mouse models of colon adenocarcinoma and squamous skin cancer found that developmental hierarchies were preserved in primary tumors and dependent upon the proliferation of stem-like cells for continued expansion (Driessens et al., 2012; Schepers et al., 2012). As we mentioned beforehand that SOX2 was closely related to the ‘lineage-specific survival mechanism’ in lung cancers, where SOX2 expression alone or together with other cooperating mutations could act as a ‘determinative switch’ in turning different cell types in lung regardless of cells of origin into LSCC or LADC (Figure 2G) (Lu et al., 2010; Mukhopadhyay et al., 2014).

These literature reviews shed light on prognostication of SOX2 as a marker and key driver for tumor aggressiveness in contexts of drug resistance and clinical relapse in addition to cancer stemness. ‘Survival of the Fittest’ SOX2 provides this fitness to the rare subpopulation of the tumor cells, enables them to persist under extreme drug selection pressures, and empowers them to cope with various therapeutic options in cancers, thereby initiating tumor regrowth and causing clinical relapse (Supplementary Figure S1).

## SOX2 as potential therapeutic targets

**Figure 3:**
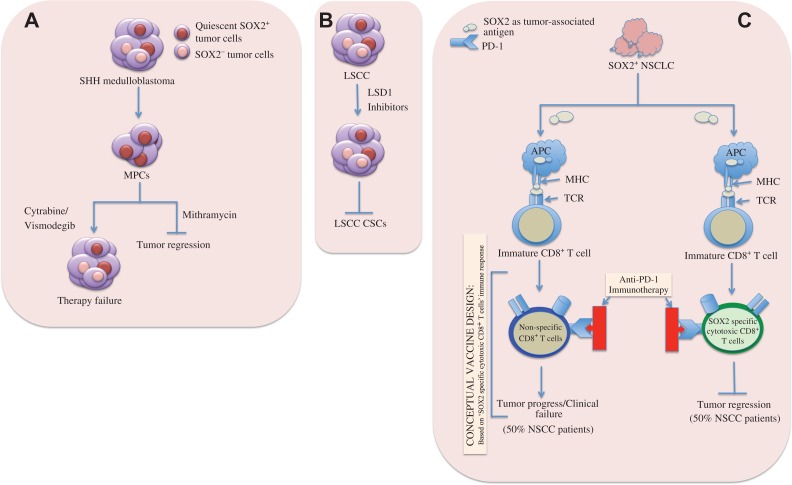
SOX2-expressing tumor cells as potential therapeutic targets. (**A**) Anti-mitotic chemotherapy (e.g. cytarabine) and sonic hedgehog (SHH) pathway inhibitor (e.g. vismodegib) fail to kill SOX2^+^ MPCs and cause CSC-mediated clinical relapse (left). Mithramycin, a highly effective drug against SOX2^+^ mouse and human SHH medulloblastoma cells, can target SOX2^+^ MPCs *in vitro* and stop tumor regrowth (right). (**B**) In LSCC, SOX2 is overexpressed due to copy number gain at 3q26.33. LSD1 is highly expressed in SOX2^+^ LSCC cells. Inhibition of LSD1 expression by LSD1 inhibitors effectively reduces SOX2 expression, thereby suppressing generation of CSCs and oncogenic potentiality of SOX2-dependent lineage-specific survival in SOX2^+^ tumors. (**C**) Potentiality of vaccine development targeting SOX2 in NSCLC patients. About 50% NSCLC patients could elicit SOX2-specific CD8^+^ T cell immune responses while the remaining 50% patients could not. SOX2-specific immune responses are amplified upon administration of anti-PD-1 immunotherapy to patients with SOX2-specific CD8^+^ T cells, thus, leading to the tumor regression. The rest of the patients lacking SOX2-specific T lymphocytes fail to respond to anti-PD-1 immunotherapy, resulting in disease relapse, and thus can constitute ideal candidates for SOX2-targeting vaccines. APC, antigen presenting cell; MHC, major histopatibility complex; TCR, T cell receptor.

Design and development of drugs targeting SOX2 can provide better therapeutic regimens because SOX2^+^ tumor cells are key player in seeding CSC and driving therapy resistance. In SHH medulloblastoma (MB), quiescent SOX2^+^ cells could cause relapse where high frequency of SOX2^+^ cells revealed an increase in therapy-resistant MPCs (e.g. cytarabine or vismodegib), a predictive signature of poor outcome in patients with SHH-medulloblastoma (Vanner et al., 2014). Targeting SOX2^+^ cells in tumor masses with mithramycin, a drug that is highly effective against SOX2^+^ mouse and human SHH MB cells *in vitro*, stopped the tumor growth (Figure 3A) (Vanner et al., 2014). Zhang et al. (2013) demonstrated that lysine-specific demethylase 1 (LSD1) was highly expressed in SOX2^+^ tumor cells and LSD1-specific inhibitors selectively retarded the growth of SOX2-expressing LSCC, while SOX2-negative cells remained unaltered. In addition, LSD1 could inhibit sensitized SOX2^+^ breast-, ovarian-, and other carcinoma cells and maintain the sensitivity to chemotherapy via its coordination with multiple epigenetic regulatory complexes (Zhang et al., 2013; Yang et al., 2018). The resultant reduction of SOX2 expression suppressed oncogenic potentiality of SOX2-dependent lineage-specific survival, thus, serving as a selective epigenetic target for therapy in SOX2^+^ cancers. Together, these studies provide impactful insights into designing anti-tumor drugs by exploiting oncogenic SOX2 as a potential candidate for therapeutic purposes (Figure 3B). In glioblastoma, SOX2-dependent subset of cells having cancer stem-like phenotypes display low or no sensitivity to mono-treatment with the PDGF receptor inhibitor (e.g. imatinib) or IGF-1 receptor inhibitor (e.g. NVP-AEW541). The resistance to PDGF- and IGF-1 receptor inhibitors was related to SOX2 expression and it could be overcome by depleting SOX2 and the approach could confer sensitivity to mono-treatment with either of these two receptor inhibitors (Hägerstrand et al., 2011). In addition, resistance to these receptor inhibitors could be hammered by combining treatment with imatinib and NVP-AEW541, which could be considered in ongoing efforts to develop novel stem cell-targeting therapies and the reason was because clinical trials with imatinib in high-grade gliomas with history of frequent tumor recurrence, in general, had failed to yield major positive results (Dresemann, 2005; Wen et al., 2006).

Although the lineage-specific ablation of SOX2^+^ tumor cells or suppression of SOX2 expression in cancers either by genetic means (Table 1) or by anti-cancer drugs (e.g. mithramycin or LSD1 inhibitors) could reduce or halt tumor growth *in vitro*, these therapeutic options need to overcome a greater challenge in cancer patients in terms of recognizing cancer and non-cancer stem cells because both of them express SOX2. Empowering the inherent ability to enhance immune defense mechanism in host could, therefore, be a better solution in this aspect. Basically, the presence of SOX2-specific T cells (Dhodapkar et al., 2013) and humoral immune responses against SOX2 had been identified in the patients with NSCLC and small cell lung cancer (SCLC) (Gure et al., 2000), respectively and this finding holds potential for immunotherapy targeting the SOX2-expressing tumors. In lung cancer, approximately 50% of a cohort of NSCLC patients elicited both CD4^+^ and CD8^+^ T-cell responses against SOX2 and the responses were readily detectable in the peripheral blood mononuclear cells. Although NSCLC regression upon immunotherapy with anti-programmed death-1 monoclonal antibodies (anti-PD-1) was associated with T-cell responses against SOX2, none of the patients who lacked SOX2-specific T cells could experience disease regression following immune checkpoint blockade and it had been shown that the administration of PD-1-blocking antibodies was associated with amplification of SOX2-specific immune responses *in vivo* (Dhodapkar et al., 2013). The link between antigen-specific T-cell immunity and clinical responses to PD-1 blockade in NSCLC patients suggests that the patients lacking T-cell responses against SOX2 may constitute ideal candidates for SOX2-targeting vaccines compared to those individuals with pre-existing anti-tumor immune responses who have already been benefited clinically from immune checkpoint-blocking agents. This also might hold the potential to develop vaccines targeting SOX2^+^ tumorigenic stem cells (Dhodapkar and Dhodapkar, 2011) (Figure 3C). In addition, Schmitz et al. (2007) identified SOX2 as a novel glioma-associated antigen and potential target for T cell-based immunotherapy. They demonstrated that selective overexpression of SOX2 in the vast majority of malignant gliomas (on both mRNA and protein levels) in contrast to the normal cortex with nearly undetectable level of SOX2, provided an advantage for T cell-based immunotherapy. Additionally, they discovered human leukocyte antigen (HLA)-A*0201-restricted SOX2-derived peptides that were capable of eliciting glioma-reactive CD8^+^ cytotoxic T lymphocyte (CTLs) responses that could destroy glioma cells. Similarly, recent finding by Vasquez et al. (2017) revealed that tumor cells, but not the surrounding normal tissue, in pediatric gliomas of all histopathological grades expressed SOX2 and the presence of T-cell immunity to SOX2 had been detected in both blood and tumor infiltrating T-cells in the children and young adults with gliomas. The CD8/CD4 T-cells with tissue resident memory (TRM) phenotype are co-expressed with several inhibitory immune checkpoints (ICP) including PD-1, PD-L1 and TIGIT. Generation of distinct subset of T-cells could, therefore, be an important target for vaccine development in glioma. Frequent co-expression of ICPs along with the TRM cells suggests that combinatorial therapies may be needed to overcome inhibitory signals in these T-cells.

## Key outstanding questions

In majority of cancers, SOX2 expression had been predominantly identified in the absence of genetic amplifications and might depend upon yet unknown upstream regulatory mechanisms (Brass et al., 1996; Balsara et al., 1997; Lengerke et al., 2011). Since epigenetic regulations principally participate in self-renewal of stem cells, it could be possible that SOX2 expressions in CSCs are triggered by some epigenetic events as exemplified by frequent hypomethylation of SOX2 promoters in GBM patients due to deregulated methylation (Alonso et al., 2011). The precise mechanism(s) behind the transcriptional reactivation of the SOX2 promoter in CSCs has yet to be elucidated. How do SOX2 expression patterns in CSCs differ from that of normal stem cells? How is contextual signal(s) from tumor microenvironment engaged in this process, and what’s about their post-translational regulation, especially in CSCs? The answers to these questions will undoubtedly develop CSC biology in terms of tumor initiation and regulatory process, which would ultimately help to develop long-lasting therapeutic strategies targeting CSCs.

A little has been known about how SOX2 promoter is turned on upon exposures to anti-cancer therapies. Elevation of SOX2 expression in tumor-propagating cells could be attributed to the fact that SOX2 promoter becomes immensely operative under stress conditions upon exposure to different anti-tumor therapies, thus, enabling them to survive under therapy-driven adverse environments and ultimately the process leads to disease relapse through generation of CSCs.

SOX2 has been identified as a tumor-associated antigen in the patients with both LSCC (Dhodapkar et al., 2013) and glioma (Schmitz et al., 2007), where SOX2-specific T cell immune responses are mounted against SOX2 and this phenomenon results in tumor regression. At present, it is not clear how SOX2 is presented on the surface of the tumors as antigen to elicit T cell responses. Disclosing the underlying mechanism could add new insights into T cell-based immunotherapy to treat SOX2-expressing tumor cells or CSCs.

Targeting SOX2^+^ cancer cells could be a strong therapeutic strategy to expunge CSCs and the approach requires bona fide study because SOX2 is also expressed in normal stem cells, and in general, SOX2^+^ tumor cells remain quiescent. Hence, it is an imperative to recognize CSCs and non-cancer stem cells for the targeted cancer therapy. Perceiving and focusing on the precise regulation of SOX2 in generation of cancer stemness and drug resistance could considerably improve the therapeutic options for the patients with a multitude of cancers, especially those with highly refractory tumors, as the ability to eradicate the tumor-initiating population is likely to be the only way to prevent recurrence.

## Supplementary Material

mjy080_Supplementary_material
