# Supplementary material for: SOX2 in cancer stemness: tumor malignancy and therapeutic potentials
Source: J Mol Cell Biol. 2018 Dec 5;12(2):85–98. doi: 10.1093/jmcb/mjy080 (PMC7109607; doi:10.1093/jmcb/mjy080)
Supplement: mjy080_Supplementary_material [file mjy080_supplementary_material.pdf]

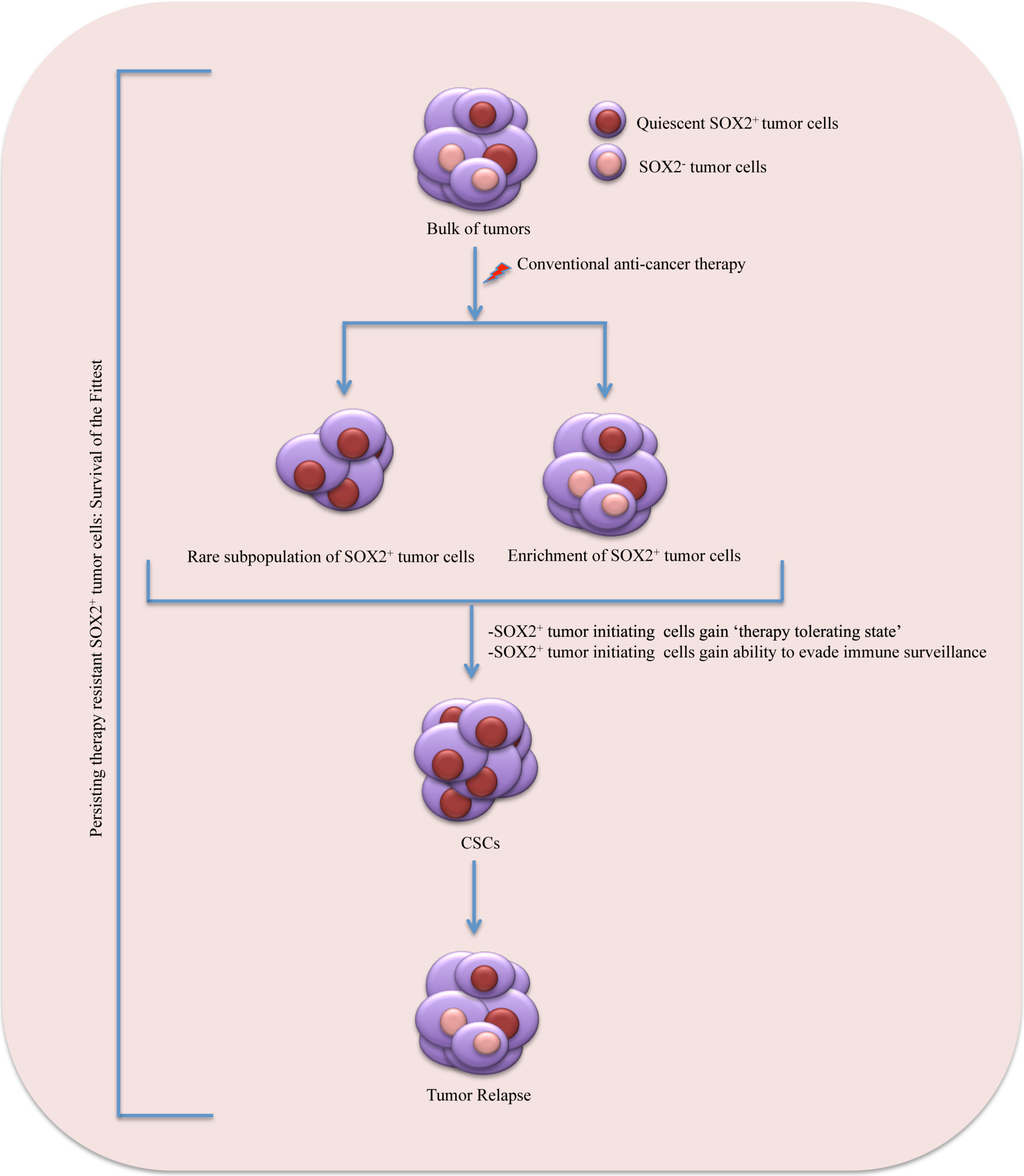

Figure S1. Therapy-resistant SOX2-expressing tumor cells as precursor for CSCs.

High SOX2 expression either due to genetic insults (3q26 amplification) or epigenetic cues from tumor microenvironment leads to generation of CSCs. Conventional chemotherapy kills most of the tumor bulks leaving behind therapy resistant, rare subpopulation of $SOX2^{+}$ tumor initiating cells that serve as precursor for CSCs (left). $SOX2^{+}$ tumor cells are also enriched following chemotherapies (e.g., tamoxifen, cytarabine, vismodegi etc.), and thus, favoring formation of CSCs (right). Elevated SOX2 expression imparts 'therapy tolerating state' to $SOX2^{+}$ tumor initiating cells achieved via increasing anti-apoptotic and/or pro-survival signaling, upregulation of ABC drug transporters, lineage plasticity and epithelial to mesenchymal transition (EMT). Higher SOX2 expression also cooperates $SOX2^{+}$ tumor initiating cells with evasion of immune system. Thus, CSCs acquire resistance to conventional anti-cancer therapies and lead to treatment failure.
